# A new species of *Dicranocentrus* (Collembola, Entomobryidae) from China with comments on the systematic position of the genus

**DOI:** 10.3897/zookeys.417.7373

**Published:** 2014-06-18

**Authors:** Guo-Liang Xu, Feng Zhang

**Affiliations:** 1 School of Geographical Sciences, Guangzhou University, Guangzhou 510006, China; 2 Department of Entomology, College of Plant Protection, Nanjing Agricultural University, Nanjing 210095, China

**Keywords:** *D. liuae* sp. n., chaetotaxy, Orchesellinae, *Heteromurus*

## Abstract

*Dicranocentrus liuae* **sp. n.** is described from the northern subtropical region of China. The new species is most similar to *D. wangi* Ma & Chen, 2007, but differs from it in the relatively shorter Ant. V, the 1+1 central macrochaetae on Abd. III, the number of chaetae on tenaculum, and the absence of dental spines. The systematic position of *Dicranocentrus* is also discussed. Present evidence, particularly S-chaetotaxy, indicates that the genus is closer to *Heteromurus* than to the unscaled species of *Orchesella* and *Orchesellides*.

## Introduction

The genus *Dicranocentrus* was erected by Schött (1893) for *Dicranocentrus gracilis*. It is characterized by 6-segmented antennae, the ratio between abdominal segments IV/III less than 2.0, scales present on antennae, legs, body, manubrium and ventral side of dens, eyes 8+8, postantennal organ absent, and mucro bidentate with a basal spine. Mari-Mutt (1979) published an excellent revision of the taxonomy, biology, and geographical distribution. Mari-Mutt (1980) divided the Orchesellinae into four tribes mainly based on the number of antennal segments, with Orchesellini (*Dicranocentrus* included), Heteromurini and Corynothrichini having 6, 5, and 4 segments, respectively. Soto-Adames (2008) did not change Mari-Mutt’s taxonomical framework, but simply added two new small tribes. However, recent molecular phylogeny of the Entomobryidae (Zhang et al. 2014a) placed *Dicranocentrus* together with *Heteromurus* in a separate clade, apart from the unscaled taxa (*Orchesella*/*Orchesellides*).

**Figure 1.**
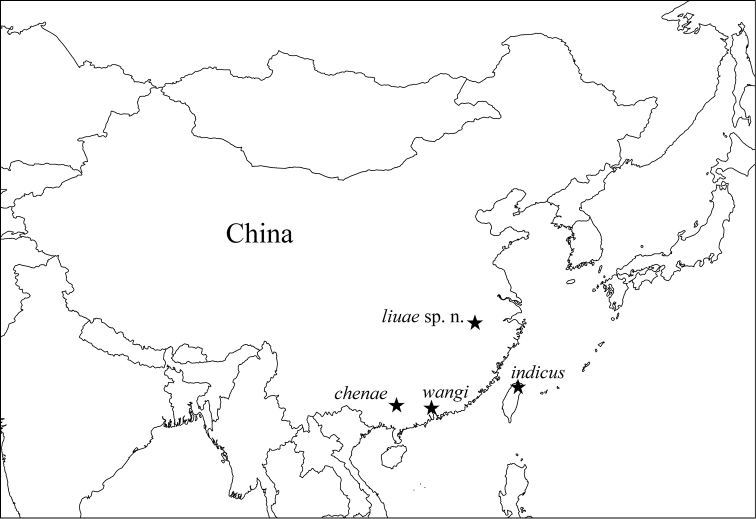
Geographical distribution of *Dicranocentrus* species from China.

So far, three *Dicranocentrus* species have been reported from China: *Dicranocentrus indicus* Bonet, 1930 from Taiwan, *Dicranocentrus chenae* Ma, Chen & Soto-Adames, 2006 from Guangxi, and *Dicranocentrus wangi* Ma & Chen, 2007 from Gangdong (Fig. 1). Here, we describe a new species from the northern subtropical region of China, compare it with other orchesellids, and discuss the systematic position of the genus.

## Materials and methods

Specimens were mounted in Marc André II solution after clearing in lactic acid and were studied using a Nikon E600 and SMZ-1000 microscope. Photographs were enhanced with Photoshop CS2/PC (Adobe Inc.). The number of macrochaetae is given by half-tergite in the descriptions. Dorsal cephalic chaetotaxy and interocular chaetae follow Mari-Mutt (1979, 1986). Types are deposited in the collections of the Department of Entomology, College of Plant Protection, Nanjing Agricultural University (NJAU), P. R. China.

Abbreviations. Th. I–III – thoracic segment I–III; Abd. I–VI – abdominal segment I–VI; Ant. I–IV – antennal segment I–IV; mac – macrochaeta/ae; mic – microchaeta/ae; ms – S-microchaeta/ae; sens – ordinary tergal S-chaeta/ae; post-labial quadrangle – PLQ.

## Taxonomy

### Dicranocentrus liuae sp. n.

Taxon classificationAnimaliaEntomobryomorphaEntomobryidae

http://zoobank.org/E9501B65-B6EC-4D84-AC1C-1BC07A65E5B8

Figs 2 – 14 , Table 1

#### Type locality.

China, Anhui, Shitai, Guniujiang, 30.092°N, 117.482°E, altitude 208m.

#### Material.

Holotype: ♀ on slide, China, Anhui, Shitai, Guniujiang, the entrance of Yan hamlet, 30.092°N, 117.482°E, altitude 208m,15 August 2011, F Zhang, DY Yu and YH Ren leg. (#C9676). Paratypes: 3 ♀♀ and 1 ♂ on slides, 4 in alcohol, same data as holotype. Other material: 1 ♀ on slide, China, Anhui, Shitai, Gongxi, 27 August 1994, JX Chen leg. (#C8391); 1 ♀ on slide, China, Zhejing, Jin Xian, Tiantongshan National Natural Reserve, 10 June 1995, JX Chen leg. (#C8458).

#### Description.

Body length up to 1.8 mm.

**Figure 2.**
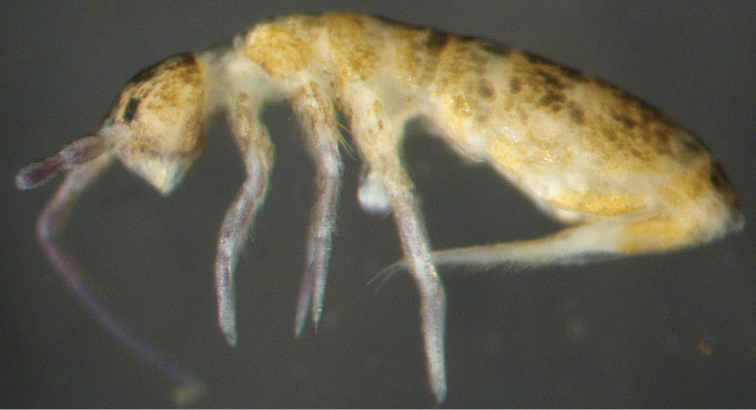
*Dicranocentrus liuae* sp. n. Habitus.

Ground colour pale yellow. Eye patches dark. Blue pigment present on antennae and legs (Fig. 2). Scales brown, rounded, truncate, or pointed with numerous short striations; scales present on Ant. I‒IV, body, legs, both side of ventral tube and manubrium, and ventral side of dens.w

**Figures 3−10.**
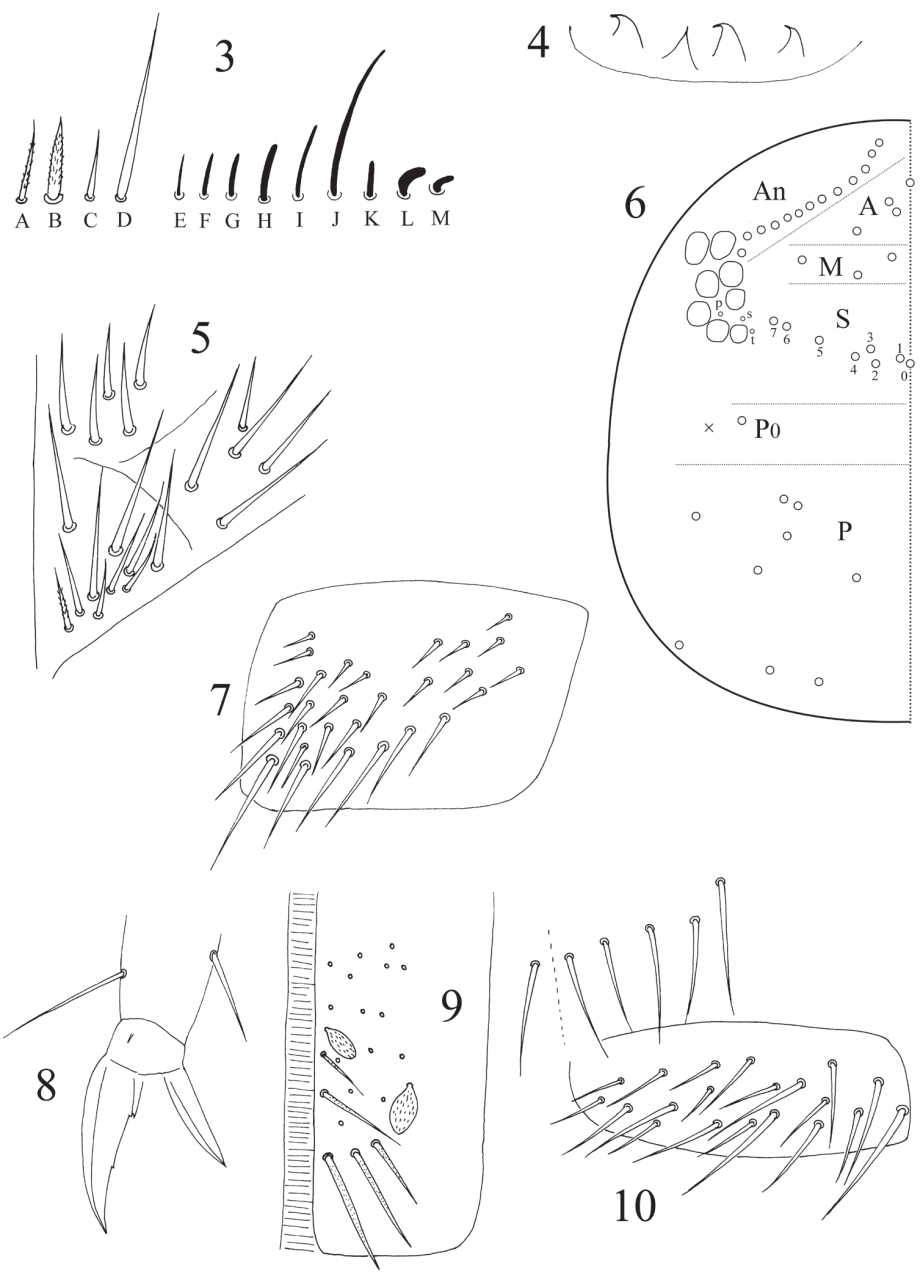
*Dicranocentrus liuae* sp. n. **3** antennal chaetae **4** labral papillae **5** labial chaetae **6** dorsal cephalic chaetotaxy **7** trochanteral organ **8** hind claw **9** anterior face of ventral tube **10** distal posterior and lateral flap chaetae of ventral tube.

Antenna 2.0–2.7 times as long as cephalic diagonal. Ant. V and VI annulated and their length ratio as 1.2–0.7: 1. Four types of common chaetae observed: ciliate, thin (Fig. 3A) or thick (Fig. 3B) mic; smooth, straight, spiny mic on Ant. I and III (Fig. 3C); long, straight chaetae smooth or weakly ciliate (Fig. 3D). Most S-chaetae slightly curved, short (Fig. 3E–I) or long (Fig. 3J). Distal Ant. II with 1 rod-like S-chaeta ventrally (Fig. 3K), 2 strongly curved, thickened S-chaetae externally (Fig. 3L–M). Ant. VI apical bulb absent.

Eyes 8+8, G and H smaller. Prelabral and labral chaetae 4/5, 5, 4, all smooth; prelabral ones stronger than labral ones. Labral papillae 4, cone-like with apical spine often curved (Fig. 4). Mandibles with 4+5 apical teeth. Lateral process of labial palp papillae E as thick as normal chaetae, with tip not reaching apex of labial papilla. Subapical chaeta of maxillary outer lobe thicker than apical; 3 smooth sublobal hairs on maxillary outer lobe. Labium with 5 smooth proximal chaetae and 8–10 submentum chaetae; the most external one A_5_ on mentum shorter than A_1‒5_; at most 1 ciliate chaeta on submentum (Fig. 5). PLQ chaetae smooth and 2+2 weakly ciliate chaetae posterior to PLQ. Dorsal cephalic chaetotaxy with10–13 antennal (An), 3 median (M), sutural S_0–7_, 1 postocular (P_0_) and 9 posterior (P) mac. Interocular chaetae 3 as p, s, t (Fig. 6).

Trochanteral organ with about 30 smooth spiny chaetae (Fig. 7). Some inner differentiated tibiotarsal chaetae smooth under light microscope. Unguis with 3 inner teeth, all minute. Unguiculus lanceolate with outer edge smooth. Tenent hairs acuminate (Fig. 8).

**Figures 11−14.**
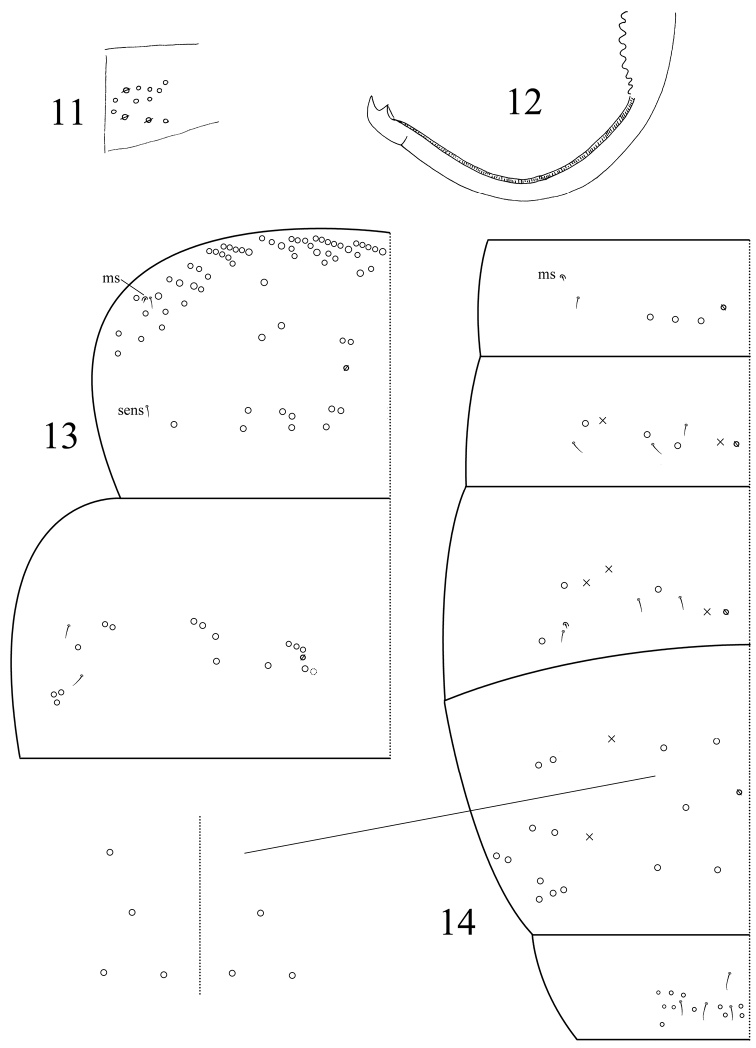
*Dicranocentrus liuae* sp. n. **11** manubrial plaque **12** mucro **13** thoracic chaetotaxy **14** abdominal chaetotaxy, inset, variation in number of inner mac.

Abd. IV 1.66–1.89 times as long as Abd. III along dorsal midline. Ventral tube anteriorly with many weakly ciliate chaetae and some scales (Fig. 9); posteriorly with many smooth chaetae; both sides with scales; each lateral flap with about 20 smooth chaetae (Fig. 10). Tenaculum with 4+4 teeth, corpus with 2–4 smooth chaetae. Manubrial plaque with 3 pseudopores and 5–9 ciliate chaetae on each side (Fig. 11). Manubrium dorsally with rows of smooth chaetae but their number not clear. Dens without inner spines. Dental of lobe with 1+1 large blunt ciliate and about 5+5 small smooth chaetae. Smooth distal part of dens 4.4–5.7 times as long as mucro; mucro bidentate with two subequal teeth (Fig. 12).

Th. II with 2 inner and 2 outer mac on medial area, 9 posterior mac, 1 ms and 2 sens; anterior ms external to sens. Th. III with 15–16 mac and 2 lateral sens (Fig. 13). Abd. I with 3 mac, 1 ms and 1 sens; ms external to sens. Abd. II with 2 inner, 1 lateral mac and 3 sens. Abd. III with 1 inner, 2 lateral mac, 1 ms and 3 sens. Abd. IV with 3–5 inner, 10 lateral mac, and many (number undetermined) elongate sens. Abd. V with 4 sens (Fig. 14).

#### Etymology.

Named after the former member Ms L. Liu in our lab, who initiated the study of the genus in China.

#### Ecology.

In litter or on leaves of forest floor.

#### Remarks.

This new species belongs to *sundanensis*-group according to Mari-Mutt(1979). It is the only member with 1+1 inner mac on Abd. III in *sundanensis*-group. It is most similar to *Dicranocentrus wangi* in labrum, cephalic chaetotaxy, trochanteral organ, tergal chaetotaxy of thorax and Abd. I–II, ventral tube, and claw structure. It differs from the latter in having a shorter Ant. V, 9 posterior cephalic mac, 1+1 inner mac on Abd. III, 2–4 chaetae on tenaculum, and the absence of dental spines (Table 1). S-chaetotaxy is also described in the genus for the first time: ms 1, 0|1, 0, 1, 0, 0 and sens 2, 2|1, 3, 3, ?, 4.

**Table 1.** Comparison between *Dicranocentrus liuae* sp. n. and *Dicranocentrus wangi*.

Characters	*Dicranocentrus liuae* sp. n.	*Dicranocentrus wangi*
Ratio of Ant. V/VI	0.7–1.2	1.2−2.3
Posterior cephalic mac	9	5
Lateral flap of ventral tube	about 20	28−50
Chaetae on tenaculum	2−4	4−10
Dental spines	absent	present
Inner mac on Abd. III	1	2

## Discussion

Mari-Mutt (1979) considered that the closest relatives of *Dicranocentrus* were *Orchesella* and *Dicranorchesella* because the three genera shared 6-segmented antennae. None questioned the systematic position of *Dicranocentrus* before the work of Zhang et al. (2014a), who also discussed the disputable use of secondary and unstable structures (such as number of antennal segments and number of chaetae on trochanteral organ) during development in modern taxonomy. Taking no account of antennae, *Dicranocentrus* shares most characters with *Heteromurus*: the presence of the same type of body scales, pigment reduced or scattered on the body, relatively fewer tergal macrochaetae, dental spines often present, and 3 ordinary S-chaetae on Abd. II/III (see also *Heteromurus nitidus*, Szeptycki 1979). An additional middle ordinary S-chaeta compared to those on species belonging to the Entomobryini/Willowsiini implies that both *Dicranocentrus* and *Heteromurus* are possibly closer to the Entomobryinae *sensu* Szeptycki, 1979, than previously thought. The pattern of four S-chaetae on Abd. V in *Dicranocentrus* is also similar to that of *Heteromurus* with the latter lacking the middle one. Compared to *Dicranocentrus*/*Heteromurus*, *Orchesella*/*Orchesellides* have much more abundant macrochaetae on each tergum and S-chaetae (usually >5) on Abd. II, III and V. The idea that the presence of body scales is a synapomorphy in *Dicranocentrus*/*Heteromurus* was strongly supported by molecular phylogeny, although body scales cannot be assumed to be a synapomorphy of the scaled genera (Willowsiini) of Entomobryinae by Zhang et al. (2014b). Body scales have been used successfully to define many groups, such as the Tomoceridae, Oncopoduridae, Seirinae, Lepidocyrtinae, and Cyphoderinae. Mari-Mutt (1979) proposed that *Dicranocentrus* originated from *Orchesella* via an intermediate stage represented by *Dicranorchesella* (with short ciliated chaetae and scales present). Mari-Mutt (1979) stated that *Dicranorchesella*, which has abundant cephalic and tergal macrochaetae, is quite close to *Orchesella*. However, of the pointed and fusiform scales of *Dicranorchesella* indicate that it represents a lineage independently derived from *Dicranocentrus*; its relationship with *Orchesella* possibly resembles that of *Willowsia*/*Entomobrya* as shown by Zhang et al. (2014a, b). A systematic review and phylogeny based on larger samples would ultimately resolve the systematic position of *Dicranocentrus*.

## Supplementary Material

XML Treatment for Dicranocentrus liuae
